# Astrocytic Mitochondrial Membrane Hyperpolarization following Extended Oxygen and Glucose Deprivation

**DOI:** 10.1371/journal.pone.0090697

**Published:** 2014-02-28

**Authors:** Andrej Korenić, Johannes Boltze, Alexander Deten, Myriam Peters, Pavle Andjus, Lidija Radenović

**Affiliations:** 1 Centre for Laser Microscopy, Department of Physiology and Biochemistry, Faculty of Biology, University of Belgrade, Belgrade, Serbia; 2 Fraunhofer Institute for Cell Therapy and Immunology, Leipzig, Germany; 3 Translational Centre for Regenerative Medicine, University of Leipzig, Leipzig, Germany; 4 Massachusetts General Hospital and Harvard Medical School, Boston, Massachusetts, United States of America; Univ. Kentucky, United States of America

## Abstract

Astrocytes can tolerate longer periods of oxygen and glucose deprivation (OGD) as compared to neurons. The reasons for this reduced vulnerability are not well understood. Particularly, changes in mitochondrial membrane potential (Δψ_m_) in astrocytes, an indicator of the cellular redox state, have not been investigated during reperfusion after extended OGD exposure. Here, we subjected primary mouse astrocytes to glucose deprivation (GD), OGD and combinations of both conditions varying in duration and sequence. Changes in Δψ_m_, visualized by change in the fluorescence of JC-1, were investigated within one hour after reconstitution of oxygen and glucose supply, intended to model *in vivo* reperfusion. In all experiments, astrocytes showed resilience to extended periods of OGD, which had little effect on Δψ_m_ during reperfusion, whereas GD caused a robust Δψ_m_ negativation. In case no Δψ_m_ negativation was observed after OGD, subsequent chemical oxygen deprivation (OD) induced by sodium azide caused depolarization, which, however, was significantly delayed as compared to normoxic group. When GD preceded OD for 12 h, Δψ_m_ hyperpolarization was induced by both GD and subsequent OD, but significant interaction between these conditions was not detected. However, when GD was extended to 48 h preceding OGD, hyperpolarization enhanced during reperfusion. This implicates synergistic effects of both conditions in that sequence. These findings provide novel information regarding the role of the two main substrates of electron transport chain (glucose and oxygen) and their hyperpolarizing effect on Δψ_m_ during substrate deprivation, thus shedding new light on mechanisms of astrocyte resilience to prolonged ischemic injury.

## Introduction

Oxidative phosphorylation is the major adenosine triphosphate (ATP) synthesis pathway in mitochondria. Energy required to drive this process is stored as the mitochondrial membrane potential (Δψ_m_) across the inner mitochondrial membrane. This is realized by respiratory proton (H^+^) pumps as core elements of the electron transport chain (ETC, complexes I–IV). Lack of the two main substrates of the ETC, glucose (electron donor) and oxygen (electron acceptor), causes a breakdown of the vital trans-membrane potential and thereby ceases oxidative phosphorylation.

Since the brain lacks sufficient oxygen and glucose storage capabilities, interruption of continuous blood supply to the organ *in vivo* (cerebral ischemia) can lead to disastrous consequences on the cellular level already within seconds to minutes, and causes macroscopic brain infarction in the long run. However, there are remarkable differences regarding the tolerance of ischemic conditions among the major brain cell populations. In contrast to neurons, astrocytes are more resistant to ischemic conditions *in vitro* than *in vivo*. The cells were reported to be relatively resistant to sole oxygen deprivation (OD) or glucose deprivation (GD) when compared to combined oxygen-glucose deprivation (OGD). Less than 3 h of OGD do not produce irreversible astrocyte injury [1], [2], [3] and signs of apoptosis in astrocytes have been reported not earlier than after 4 h of OGD [4]. Exposures longer than 6–8 h significantly increase the number of apoptotic and necrotic astrocytes in culture [1], [5], and induces formation of autophagosomes [6] as well as peaking LDH release [2], [7] from the remaining cells.

A relatively short period of oxygen withdrawal (up to 20 min) causes mitochondrial depolarization thereby abolishing Δψ_m_ fluctuations in cultured astrocytes [8]. However, much longer exposure times are needed for increased lactate dehydrogenase (LDH) release and noticeable morphological changes indicating cellular injury, such as somatic swelling and detachment from the culture dish [9]. Findings from more recent studies suggest that astrocytes can maintain hyperpolarized mitochondria even during extended periods of OD [10]. When glucose supply is preserved, even 5 days of continuous OD cause little astroglial injury [1].

On the other hand, already 1.5 h of GD under normoxic conditions induce mitochondrial membrane hyperpolarization, followed by Δψ_m_ decrease 4 h after GD onset. Even though not immediately followed by significant LDH increase or astrocyte death [2], [11], these observations implicate different levels of tolerance to OD versus GD. However, astrocytes are also surprisingly resilient against GD: cell injury comparable to 6–8 h of OGD injury requires >36 h of GD [1]. Preceding structural cell damage, astrocytes exhibit changes in Δψ_m_ dynamics already between 45 min and 2 h of exposure to OGD. However, the exact time point of these early Δψ_m_ imbalances onset is discussed controversy [3], and changes in Δψ_m_ during simulated reperfusion after extended OGD exposures were not investigated in detail yet.

Therefore, the present study investigated Δψ_m_ during simulated reperfusion within one hour after extended exposure of cultured primary mouse astrocytes to GD or OGD. Since it has been proposed that either OD or low cytosolic ATP/ADP ratio can up-regulate glycolysis and thus support Δψ_m_ via ATP hydrolysis [12], [13], [14], we have also studied the effect of reduced glucose levels (preconditioning) prior to OGD. In addition, by applying a potent respiration inhibitor (NaN_3_) during the simulated reperfusion, we have investigated the effect of “chemical OD” on Δψ_m_ which was already affected by preceding OGD.

## Materials and Methods

### Astrocyte Primary Cell Culture

All animal experiments were performed according to the NIH Guide for Care and Use of Laboratory Animals (1985) and the European Communities Council Directive (86/609/EEC), and were approved by the responsible animal welfare authority at the Leipzig Regional Board (protocol number T22/12). All efforts were made to minimize animal suffering and to reduce the number of animals used.

Primary brain cells were prepared from the dissociated forebrain cortices of fetal BALB/c mice on gestation day 18 (E18) as previously described [15]. In brief, embryos were sacrificed by decapitation, brains were exposed, and meninges removed. Cortices were minced before gentle dissociation in DMEM supplemented with 10% (vol/vol) FBS. Cells were then seeded in 75 cm^2^ cell culture flasks (10 hemispheres per flask) and incubated at 37°C under a humidified 5% CO_2_-containing atmosphere. After 7 days *in vitro* (DIV7), flasks were vigorously shaken for several minutes to displace microglia and loosely adhered oligodendrocytes. Cells were washed with phosphate buffered saline (PBS), and medium was changed twice a week. After DIV14, the cells were trypsinized, split 1∶2, and further grown in DMEM containing 10% FBS for additional 10–14 days before being plated at a density of 5×10^4^ cells on poly-L-lysine-coated 14 mm cover slips. Cells were used in experiments 4 days later.

### *In vitro* Glucose Deprivation, Oxygen Deprivation and Oxidative Stress

**Figure 1:**
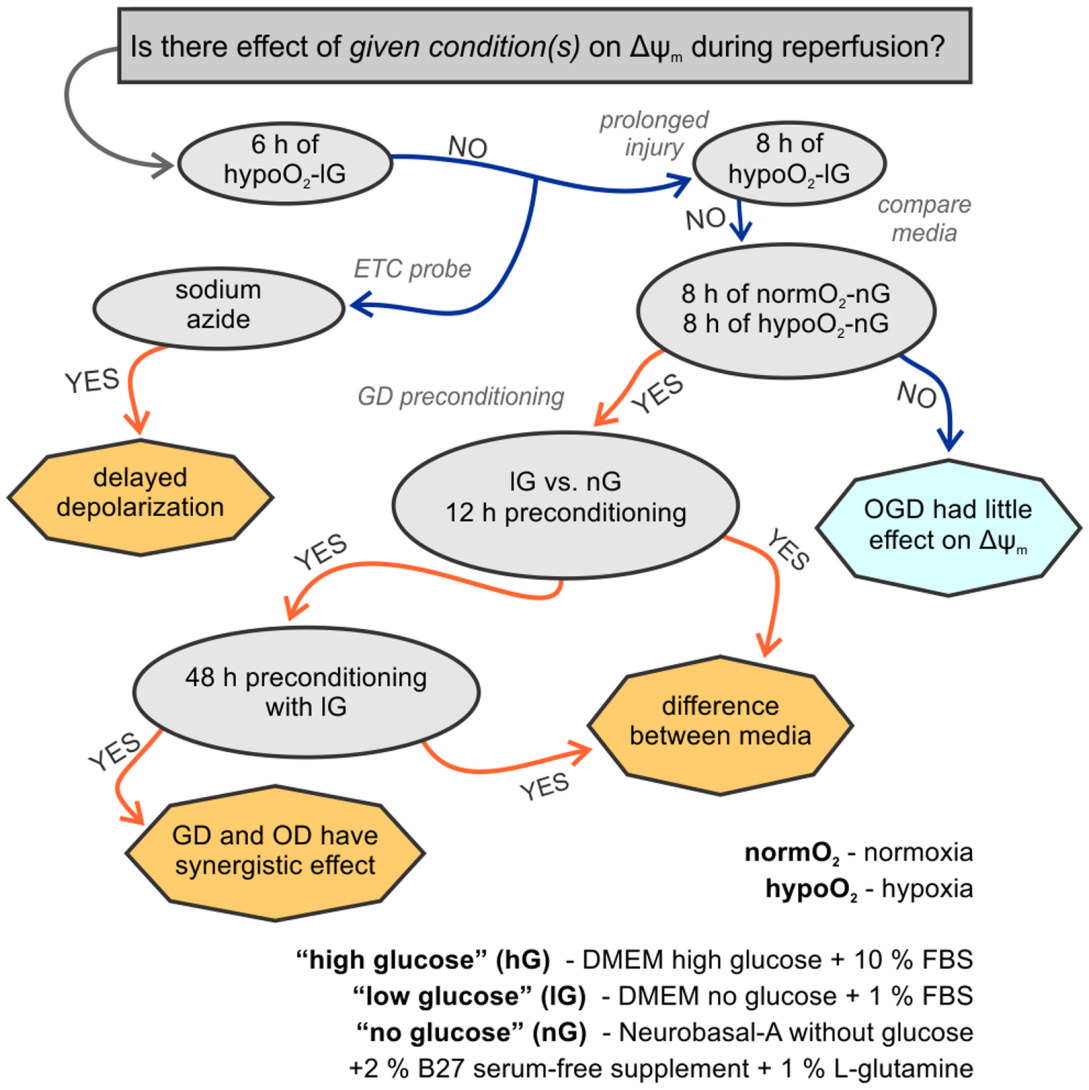
Schematic representation of the experimental design and most important results. Figure illustrates crucial steps in investigation of Δψ_m_ changes during simulated reperfusion within one hour after extended exposure of astrocytes to GD or OGD. The culturing media are designated as: high glucose (hG), low glucose (lG) or no glucose (nG). During the experiments cells were cultured either in normoxic (normO_2_) or hypoxic (hypoO_2_) conditions. Ellipsoid shapes show conditions the cells were subjected to. Text on connector lines shows whether we found significant effect of given experimental condition(s). Octagons indicate conclusions we made.

The experimental procedure was divided into three steps: glucose preconditioning in normoxic conditions (i.e. GD), OGD and reperfusion. We used three different culturing media: “high glucose” (hG), “low glucose” (lG) and “no glucose” (nG) medium (Fig. 1).

For OGD, astrocytes on cover slips were rinsed two times with incubation medium, either lG or nG. The culture medium was replaced with the incubation medium shortly before transferring plates to an incubator (Binder Labware, Tuttlingen, Germany) containing a humidified gas mixture of 1% O_2_, 5% CO_2_ and 94% N_2_ at 37°C. Control astrocyte cultures were incubated under normoxic conditions. Following OGD, cultures were reoxygenated and fresh medium was provided for 10 min at 37°C under normoxic conditions in a humidified atmosphere containing 5% CO_2,_ simulating reperfusion *in vivo*. Subsequently, astrocytes were stained for microscopic imaging (see below).

Sodium azide (NaN_3_), a potent inhibitor of the cytochrome *c* oxidase (complex IV) and F_O_F_1_-ATPase’s hydrolyzing function [16], [17], [18], [19] was used for induction of “chemical OD”. To induce mitochondrial depolarization 5, 10, or 25 mM were applied for 10 min each. We also used high concentrations of hydrogen peroxide (3 and 15 mM H_2_O_2_) to verify the results observed with NaN_3_ since such concentrations of H_2_O_2_ inflict both loss of Δψ_m_ and cell membrane integrity [20].

### Mitochondrial Membrane Potential Measurements

The mitochondrial membrane potential was examined by staining astrocytes with JC-1 (5,5′,6,6′-tetrachloro-1,1′,3,3′-tetraethylbenzimidazolyl-carbocyanine iodide), a lipophilic, cationic dye that exhibits a fluorescence emission shift upon aggregation from 530 nm (green monomer) to 590 nm (red “J-aggregates”) [14], [21], [22]. In healthy cells with high mitochondrial Δψ_m_, JC-1 enters the mitochondrial matrix in a potential-dependent manner and forms aggregates. Staining was performed using 2.5 µg/ml JC-1 at 37°C for 15 min. After staining, cells were rinsed 3x with phosphate buffered saline (PBS). Dye equilibration was allowed for 10 min at room temperature prior to imaging. Images were taken using Nikon Eclipse Ti-E inverted microscope with a Plan Fluor 10x objective. Samples were illuminated with Nikon C-HGFIE Intensilight (Precentered Fiber Illuminator) and fluorescence was recorded using Nikon DS-Qi1Mc digital camera. Stained, polarized mitochondria were detected with fluorescence settings for Cy3 (excitation/emission = 550/570 nm, EV = 50 msec). Loss of mitochondrial integrity was detected with settings for FITC (excitation/emission = 485/535 nm, EV = 500 msec). Images were analyzed using the NIS-Elements v3.0 software (Nikon Instruments Inc., Melville NY, USA) by assessing the average fluorescence intensity from an individual image. Micrographs were slightly enhanced for printing (ImageJ 1.48a) and modification parameters (brightness, contrast and LUTs) were strictly the same for each micrograph depicted in one panel to ensure comparability.

### Statistical Analysis

Measurements from individual plates were performed at least in triplicates with fluorescence intensities of all images obtained from a single experimental setting being averaged. The JC-1 fluorescence values were first normalized to its respective control for red and green signal separately before the ratio between them was calculated. All data sets were tested for normal distribution with Shapiro-Wilk’s normality test. Statistically significant differences between average values obtained in different experimental settings were analyzed by one-way ANOVA and Holm-Sidak’s multiple comparisons test as a *post hoc* test (GraphPad Prism 5.03). We used approximations of two-way ANOVA to examine the effect of culture media in comparison to OGD. All values are presented as mean ± standard deviation (SD). P-values <0.01 were considered as statistically highly significant (indicated by double symbols: *, # or Δ).

## Results

Astrocytes were exposed to GD or OGD, varying the duration and sequence of each treatment. Effects on Δψ_m_ were investigated by JC-1 staining (Fig. 1).

### Chemical OD Following OGD

Effect of chemical OD [16], [17], [18], [19] was assessed during simulated reperfusion following 6 h of OGD in lG medium. Control astrocytes were incubated under normoxic conditions in hG medium.

**Figure 2:**
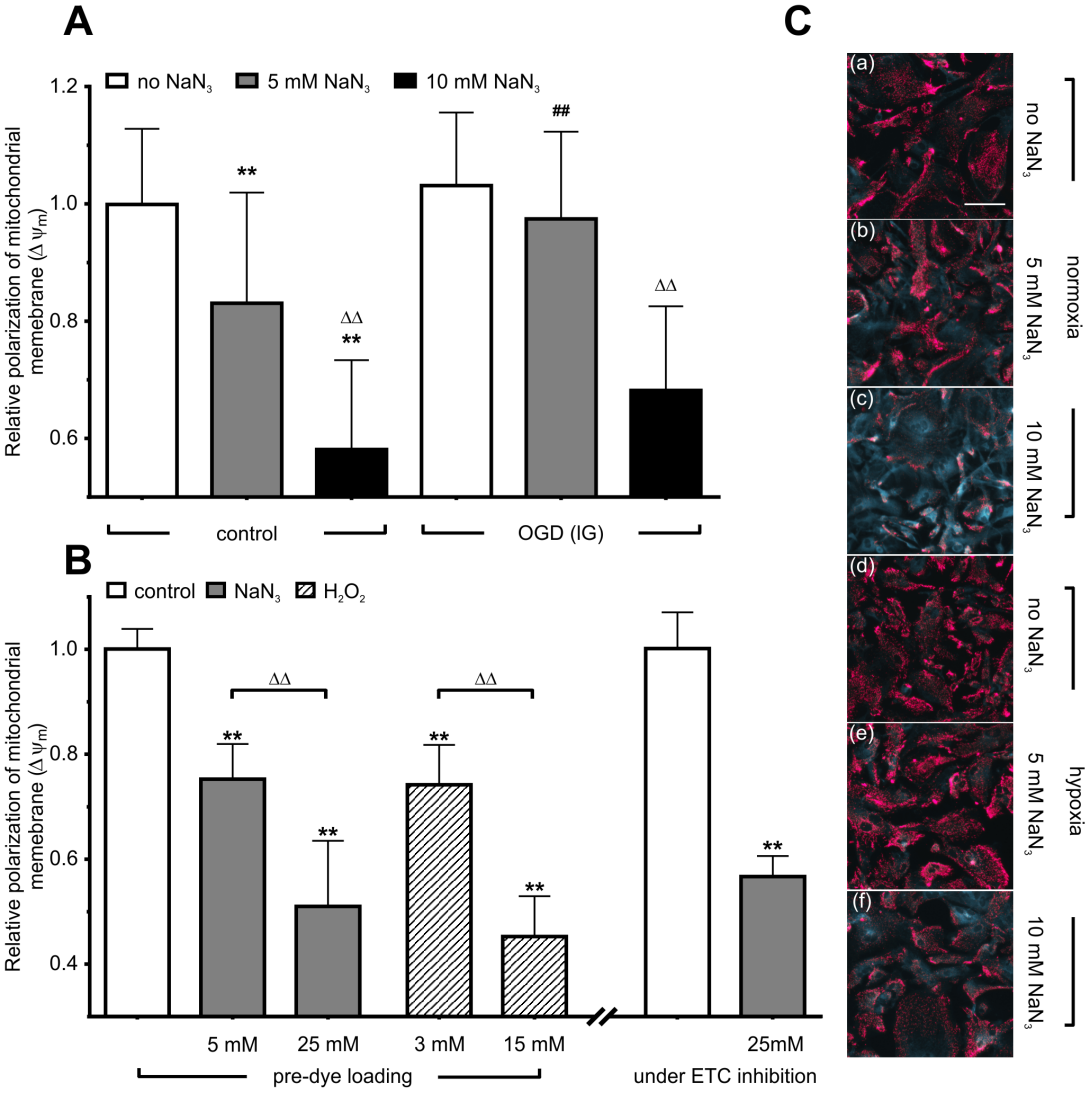
OGD in lG partly preserves Δψ_m_ (i.e. delays depolarization) caused by subsequent NaN_3_ treatment. (**A**) Sodium azide (NaN_3_) causes concentration-dependent decline of Δψ_m_ in both experimental conditions. However, OGD in lG medium significantly delayed the decrease in JC-1 fluorescence ratio caused by treatment with 5 mM NaN_3_ during simulated reperfusion. (**B**) Expectedly, both NaN_3_ and H_2_O_2_ show concentration-dependent effect by lowering the JC-1 red/green fluorescence ratio. Astrocytes were stained with JC-1 either before or during the treatment with NaN_3_ (marked as pre-dye loading and dye loading under ETC inhibition, respectively). NaN_3_ has not affected the cell membrane organization allowing at the same time JC-1 to enter the cytoplasm and mitochondria within. (**C**) Representative fluorescent micrographs of astrocytes labeled with JC-1 Original micrographs were converted to tritanope color palette (ImageJ 1.48a). Depolarization is visible in the normoxic group (b, c) (seen as concentration-dependent decrease in magenta and increase in blue color), but it is more pronounced than in OGD group after treatment with NaN_3_ (e, f). Some mitochondria remained partly depolarized. Data are expressed as a percentage normalized to the red/green fluorescence ratio values of untreated control (the first bar from left). Significant differences are indicated by **p<0.01 with respect to untreated control, ##p<0.01 between treatment and its respective control, ΔΔp<0.01 between different inhibitor concentrations.

There was no significant difference between control and OGD astrocytes (p>0.01, Fig. 2A,C). However, subsequent chemical treatment with both 5 and 10 mM NaN_3_ significantly lowered JC-1 red/green fluorescence ratio in treated normoxic astrocytes as compared to untreated control (83.1±18.9% and 58.1±15.3%, p<0.01). On the other hand, astrocytes subjected to 6 h of OGD and treated with 5 mM NaN_3_ afterwards did not show any significant change of JC-1 red/green fluorescence ratio when compared to control levels. Therefore, addition of 5 mM NaN_3_ during simulated reperfusion to both normoxic and OGD astrocytes led to significant differences between these groups (one-way ANOVA, 83.1±18.9% vs. 97.4±14.9%, p<0.01). Although normoxic astrocytes treated with 10 mM NaN_3_ exhibited lower fluorescence ratio when compared to respective OGD protocols (58.1±15.3% vs. 68.2±14.4%, respectively), there was no statistically significant difference between them.

### Chemical OD *vs.* Oxidative Damage: Effect of NaN3 *vs*. H2O2

Our next aim was to compare the effect of NaN_3_ and H_2_O_2_ on Δψ_m_ (Fig. 2B). To this end, astrocytes were exposed to different NaN_3_ or H_2_O_2_ concentrations for 15 min in order to simulate conditions of ETC impairment or oxidative stress/damage, respectively. In all experimental conditions the JC-1 fluorescence was recorded without washing out NaN_3_ or H_2_O_2_ (i.e. without simulated reperfusion), since the effect of NaN_3_ was found transient and reversible already 15 min after washing out the inhibitor in previous experiments (data not shown). A dose-dependent, significant decrease of JC-1 red/green fluorescence ratio was observed for both NaN_3_ (75.25±6.7% at 5 mM and 51±12.51% at 25 mM, p<0.01) and H_2_O_2_ (74.17±7.6% at 3 mM, and 45.3±7.7% at 15 mM; p<0.01). When red and green signals were analyzed separately, we found that NaN_3_ increased solely the green signal to 188.6±25.4% (red signal remained 98.9±21.1), while H_2_O_2_ decreased the green signal to 90.0±33.3% and also decreased the red signal to 39.8±13.5%. Loading the dye under ETC inhibition did not show any significant difference in JC-1 red/green fluorescence ratio when compared to pre-dye loading procedure.

### The Role of Glucose in Oxygen Deprivation

**Figure 3:**
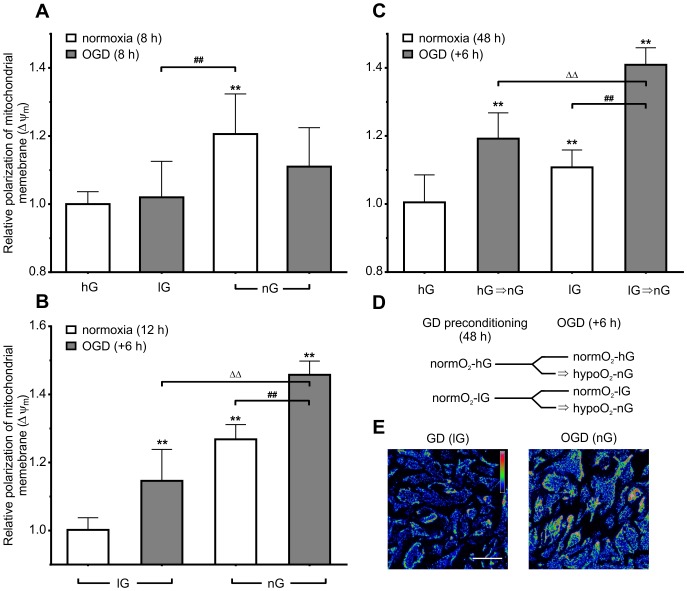
Glucose deprivation leads to hyperpolarization of Δψ_m_ after both normoxic and OGD conditions. (**A**) Glucose deprivation for 8 h increases the ratio of JC-1 fluorescence. This effect was not detected when combining GD and OD, but level of significance was noticable low (p = 0.059). (**B**) Preconditioning for 12 h with media containing reduced or no glucose promotes hyperpolarization after OGD during simulated reperfusion. After preconditioning, astrocytes were subjected to hypoxic conditions for additional 6 h (grey bars) or they were maintained in normoxic conditions (white bars). (**C**) Lowering glucose in the incubation medium leads to an increased ratio of JC-1 fluorescence during simulated reperfusion after normoxic and OGD conditions. There was significant interaction between effect of glucose in the culturing media and the effect of OGD. Astrocytes were incubated for two days either in hG or lG medium. Subsequently, OGD was conducted for 6 h (grey bars). (**D**) Schematic representation of the experimental design of C. The duration of each step is shown in brackets. (**E**) Representative red signal from fluorescent micrographs of astrocytes labeled with JC-1. Original micrographs were converted to rainbow pseudocolor pallete using LUTs (ImageJ 1.48a). Increase in red fluorescence is observed when astrocytes are incubated in OGD in nG medium as compared to normoxic conditions in lG medium (Fig. 2B). The scale bar represents 100 µm. Data are expressed as a percentage normalized to the JC-1 red/green fluorescence ratio values of untreated control astrocytes (first bar on the left). Significant differences are indicated by **p<0.01 with respect to control (normoxia in hG for Fig. 3A and 3C, normoxia in lG for Fig. 3B), ##p<0.01 between normoxia and OGD (in lG or nG), ΔΔp<0.01 between two OGD treatments.

In order to further investigate the effect of OGD on Δψ_m_, we subjected astrocytes to 8 h of OGD in either lG or nG medium, as well as solely GD in normoxic conditions (Fig. 3A).

There was no statistically significant interaction between effects of culturing media and OGD treatment (two-way ANOVA, p>0.05). OGD alone did not exert significant effects with level of significance being noticable low (p = 0.059), but the effect of culturing media alone did show significance (p<0.01, accounting for about 34% of the total variance). There was a significant increase in JC-1 red/green fluorescence ratio from the control in astrocytes cultured for 8 h in normoxic conditions in nG medium (one-way ANOVA 120.6±11.8%, p<0.01). Astrocytes cultured in nG medium and subjected to 8 h of OGD also showed a slight increase in fluorescence ratio (111.0±11.4%), however, this difference was not statistically significant (p>0.01). Astrocytes cultured in lG conditions and subjected to 8 h of OGD, also did not show any statistically significant change of fluorescence ratio (102.0±10.6%, p>0.01).

### GD Preconditioning

Since there was no significant difference in Δψ_m_ during reperfusion between astrocytes subjected to 8 hours of OGD either in lG or nG (Fig. 3A), the next set of experiments investigated the effect of OGD preceded by a GD (lG or nG) preconditioning step for 12 h (Fig. 3B,D).

There was no significant interaction between effects of preconditioning and OGD on JC-1 red/green fluorescence ratio (two-way ANOVA, p>0.05), but there were statistically significant differences between all tested groups (one-way ANOVA, p<0.01). Astrocytes that were cultured in lG medium and subjected to 6 h of OGD showed a significant increase in JC-1 red/green fluorescence ratio as compared to astrocytes incubated under normoxic conditions (114.6±9.2%, p<0.01). Incubation in nG also showed a significant increase in fluorescence ratio as compared to astrocytes incubated in lG (126.8±4.3%, p<0.01). In addition, astrocytes incubated in nG subjected to 6 h of OGD showed a further increase of fluorescence ratio (145.8±4.0%) which was significantly different from all three previously mentioned conditions (p<0.01).

### Extended Preconditioning

Further, the effect of extending preconditioning was studied. Astrocytes in normoxic conditions that were incubated for two days in lG showed significant increase of JC-1 red/green fluorescence ratio when compared to control astrocytes cultivated for 48 h in hG (one-way ANOVA, 110.8±5.1%, p<0.01; Fig. 3C). Astrocytes that were incubated in hG medium and later subjected to 6 h of OGD also showed significant increase of fluorescence ratio as compared to normoxic culture conditions (119.2±7.6%, p<0.01). The highest increase in JC-1 red/green fluorescence ratio was measured in the OGD group that was preconditioned in lG medium (140.9±5.0%, p<0.01 as compared to all other conditions). In addition, there was a statistically significant interaction between the effect of incubation media and the effect of OGD (two-way ANOVA, p<0.05, accounting for approx. 3% of the total variance), reflecting different effects of 6 h of OGD after 2 days of incubation in hG as compared to lG.

## Discussion

Astrocyte resilience to extended exposures to OGD remains controversial from the aspect of changes in mitochondrial function, especially in terms of Δψ_m_. Often preceding cell death, such changes were described for time frame between 45 min and 2 h, as well as during simulated reperfusion. Surprisingly results include both prolonged depolarization [3] and no apparent changes in mitochondrial respiratory function [23], with possibility the latter being followed by altered sensitivity to subsequent chemical treatments [24]. Taking into account that cell death gradually increases from 6 to 12 h of exposure [1], [5] we investigate changes in Δψ_m_ after 6 h exposure to OGD. We hypothesize that in those studies cells that survived the OGD insult maintained their cellular respiration and energy production after reperfusion, which is in congruence with results of vitality assays used in those experiments.

In our study there was no significant effect on Δψ_m_ within the first hour after 6 h of OGD in lG medium. To reveal the effect of OGD, we applied NaN_3_ to inhibit ETC function during the reperfusion phase. OGD in lG partly preserved Δψ_m_ (i.e. delayed depolarization) caused by treatment with 5 mM NaN_3_ during simulated reperfusion. This highly interesting phenomenon may be related to NaN_3_ inhibition of the hydrolyzing function of the F_O_F_1_-ATPase and differences in oxidative mitochondrial metabolism between incubation in normoxia in hG and OGD in lG. This should be addressed in further studies. In general, the effect of NaN_3_ on Δψ_m_ has been found transient and reversible [8], [25], but apart from binding to multiple sites on cytochrome *c* oxidase [26], NaN_3_ also binds to F_O_F_1_-ATPase and its sensitivity to NaN_3_ increases after bound ATP is hydrolyzed to produce bound ADP [16]. This leaves an open question whether cell’s reliance on ATP hydrolysis, when ATP/ADP ratio is high, makes Δψ_m_ dissipation by NaN_3_ quicker as compared to ETC inhibition alone when ATP/ADP ratio is low. As compared to relative fluorescence signals in H_2_O_2_ and ETC inhibition trials, it can be concluded that NaN_3_ has not affected the plasma membrane organization allowing at the same time JC-1 to enter the cytoplasm and mitochondria within. Namely, hydrogen peroxide decreases the activity of α-ketoglutarate dehydrogenase and NAD(P)H levels [11], [27], but inflicts apoptosis and necrosis at high (0.5–1.0 mM) and at very high concentrations (5–10 mM), respectively [20].

Similar results were obtained for 8 h of OGD regardless of whether astrocytes were incubated in lG or nG media. However, hyperpolarization was measurable to some extent (∼11% increase) during simulated reperfusion after OGD in nG medium. We were also able to demonstrate a significant effect of GD in normoxic conditions in nG yielding a ∼20% increase in Δψ_m_ during simulated reperfusion. First, these results corroborate data on the enhanced tolerance of astrocytes to extended oxygen and glucose deprivation (showing unchanged Δψ_m_ following OGD as compared to normoxic hG (control conditions). Second, we compared effects of DMEM and Neurobasal-A since the nG medium inhibits glial growth without usage of antimitotic agents simultaneously providing sustainable glucose- and serum-free environment, used commonly e.g. for culturing feeder layer of astrocytes in co-culture with neurons. On the other hand lG medium contains some residual glucose from 1% FBS which translates roughly to >700-fold reduction in glucose level, considered here as GD as similarly described elsewhere [28]. Also glucogenic amino acid L-glutamine provides astrocytes with a modest alternative source of energy. Therefore, we further compared effects of culturing astrocytes in these two media.

Astrocytes withstand Δψ_m_ dissipation and exhibit cell death upon treatment with complex I inhibitor rotenone regardless of the presence of glucose. Accordingly, glucose withdrawal for 6 h does not induce extensive oxidative stress and apoptosis in astrocytes, while GD is compensated by increased fatty acid oxidation [28]. Furthermore, a short-term or chronic increase in the concentration of glucose in culturing media markedly suppresses astrocytes oxidative mitochondrial metabolism, but increases the glycogen level and lactate release [29], [30]. These data taken altogther raise the question whether OGD has a different effect on cells with lower vs. higher oxidative mitochondrial metabolism. Additionally, hyperpolarization has been observed during simulated reperfusion after 8 h of GD in the present study. Therefore, we actually matched the total duration of the GD preconditioning and OGD treatment to the time known for OD alone to evoke astrocyte injury. This time is in fact half of the time needed for GD alone [1], [9], [31]. When comparing lG and nG, we found that the differences between media compositions gave rise to measurable changes in Δψ_m_ after OGD. Both conditions led to an increase in Δψ_m_, but did not act synergistically. Those differences between media should be addressed in further studies.

Finally, we exposed astrocytes to even longer incubation (48 h) in the lG medium before subjecting them to 6 h of OGD in nG medium. Preconditioning with lG medium led to an increase in Δψ_m_ during reperfusion affecting both normoxic and OGD group. Hyperpolarization was also observable after the OGD in nG medium following culturing in hG medium. These results suggested that lowering glucose in the culture medium several hours prior to OGD promotes hyperpolarization during the simulated reperfusion in astrocytes. Results also point to a significant interaction between preconditioning with GD and following OGD, implicating synergistic effects of both conditions in that sequence on Δψ_m_, as well as further stressing out the difference between lG and nG media.

Although lG alone didn’t exhibit any effect on Δψ_m_ (both as GD and OGD), its effect became apparent when cells were cultured for 48 h in lG medium and then subjected to 6 h of OGD in nG medium. This led to the highest mitochondrial hyperpolarization observed. Such increase in Δψ_m_ has been demonstrated following the lack of either oxygen or glucose as the main substrates of the ETC [10], [11], [32]. Hyperpolarization itself stalls the function of ETC and has been referred to as a mechanism contributing to resistance to apoptosis [12], [33]. This phenomenon is present interestingly even in damaged mitochondria [34].

In conclusion, in all experiments astrocytes showed tolerance to extended periods of oxygen and glucose deprivation regardless of whether lG or nG medium was used. We confirmed that GD is a major contributor to the increase in Δψ_m_ while the effect of OGD on Δψ_m_ is limited and, to some extent, reversible. Although latter finding seams rather peculiar, it is in line with current literature data on enhanced astrocytic tolerance to such insult. Only 14% of cells die after 6 h of OGD and that number increase to 24% after 9 h of OGD (measured 24 h after the insult) [5]. The hyperpolarization level during reperfusion phase, within one hour after exposure to OGD, was related to lowering glucose several hours prior to OGD. The effects of the two conditions, GD and OGD, showed significant interaction, while a relative hyperpolarization of the OGD cells could also be seen during the reperfusion phase following subsequent application of NaN_3_. These finding may contribute to understand the impact of cerebral ischemia on astroglial populations and to the evaluation of results, related to the glucose level and glucose deprivation, obtained by studying ischemic injury *in vitro*. Astrocyte energy metabolism seems to be directly influenced by the level of glucose. Ultimately, these results point to the protective capacity of mitochondrial hyperpolarization against apoptotic cell death, and shed light on astrocytic resistance to prolonged ischemic injury.
